# Retinal Detachment and Temporal Artery Dissection: An Elusive Case of Giant Cell Arteritis

**DOI:** 10.7759/cureus.16703

**Published:** 2021-07-28

**Authors:** Hina Farrukh, Christopher VandenBerg, Elisabeth Ertel, Heidi Griffith, Brendan Kelly

**Affiliations:** 1 Internal Medicine, AtlantiCare Regional Medical Center, Atlantic City, USA; 2 Department of Medicine, Temple University, Philadelphia, USA

**Keywords:** giant cell arteritis, retinal detachment, temporal artery dissection, temporal arteritis, corticosteroids, vision loss, pyrexia of unknown origin, retinal vasculitis, doppler us, vitreous hemorrhage

## Abstract

Giant cell arteritis (GCA), also known as temporal arteritis, is the most common systemic inflammatory vasculitis occurring in the elderly. Patients usually present with vision loss, headache, jaw claudication, diplopia, myalgia and constitutional symptoms. The most common ocular manifestations are anterior ischemic optic neuropathy (AION), choroidal ischemia and central retinal artery occlusion. Here we describe a case of GCA presenting with vision changes secondary to retinal detachment and vitreous hemorrhage. Upon temporal artery biopsy, GCA was confirmed and it was found to be accompanied by temporal artery dissection as well. Patient was treated with pulse dose intravenous steroids after which her symptoms improved. Based on our literature review, this is the first reported case of GCA presenting in this manner.

## Introduction

Giant cell arteritis (GCA) is the most common primary vasculitis in adults. To date, no definite ways of stratifying risk factors for permanent visual loss in GCA have been established. Age, hypertension, thrombocytosis, jaw claudication, and other features have been proposed as risk factors with age being the strongest one. The disease almost exclusively occurs after the age of 50 years, and its incidence peaks between the ages of 70 to 79 years [1]. Histopathologically, GCA is characterized by a nodular granulomatous inflammation of medium and large-sized arteries [2]. Although arteritic anterior ischemic optic neuropathy (AION) is the most common manifestation, GCA can affect the entire visual pathway from the retina to the occipital lobe [3]. Retinal detachment and temporal artery dissection are very rare manifestations of GCA as described below in our patient’s case. When GCA is suspected, treatment with corticosteroids is indicated on an urgent basis, as further vision loss and fellow eye involvement are usually preventable [4].

## Case presentation

**Figure 1:**
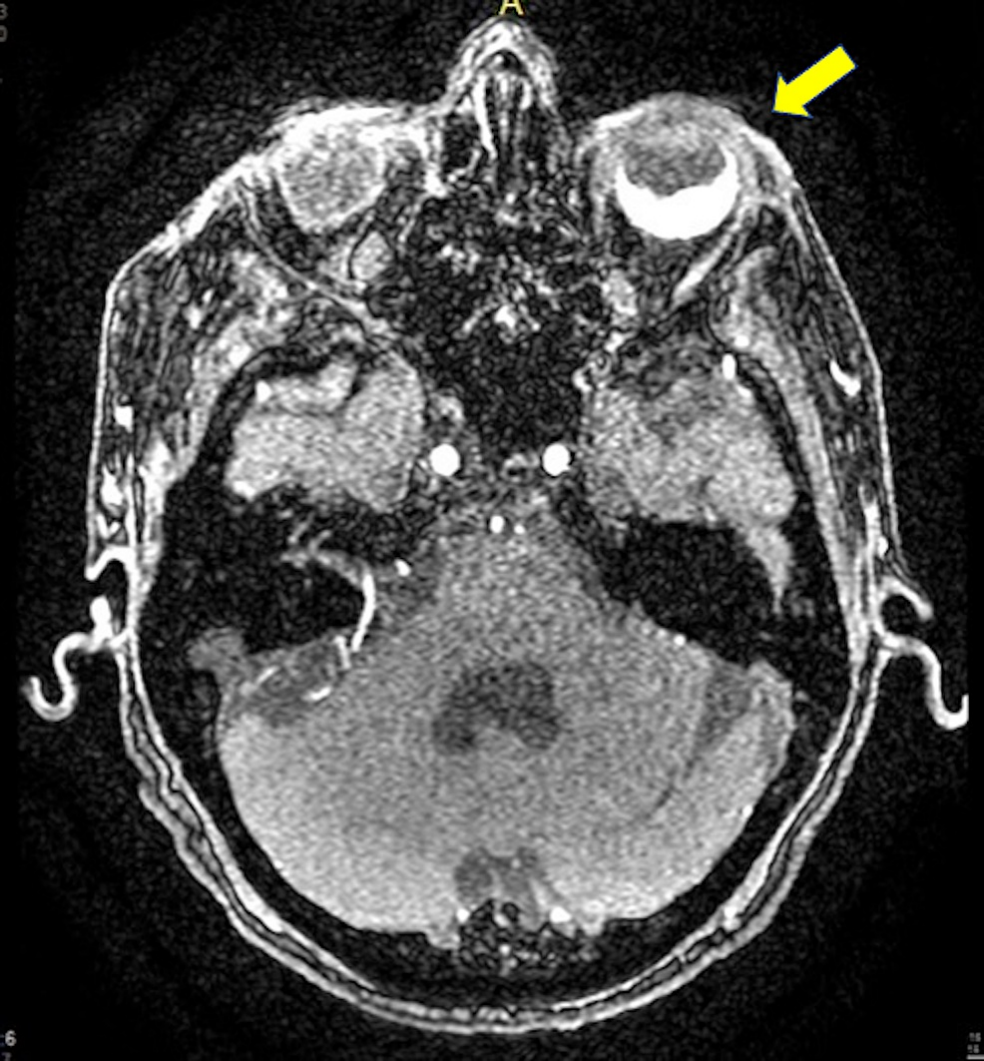
Computed Tomography (CT) of the head (without contrast) demonstrating retinal detachment with evidence of high attenuation material within the left ocular globe

A 75-year-old female with no significant past medical history presented to the emergency department with visual disturbances and ongoing fever. She reported having “snowy vision” in her left eye for one day and low-grade fever for one week associated with intermittent headaches. Upon examination she was found to be febrile and tachycardic with oral temperature of 100.7-degree Fahrenheit (F). Her blood pressure was 140/80 mmHg. Ophthalmologic examination demonstrated intact extra-ocular movements. Her pupils were equal and reactive without afferent pupillary defect. Visual acuity was 20/50 on the right side and only hand motion on the left. Visual fields had similar findings. Fundoscopy showed vitreous hemorrhage in the left eye obscuring the optic disc. Bedside ultrasound of the eye suggested retinal detachment. Computed Tomography (CT) head without contrast demonstrated retinal detachment as well with evidence of high attenuation material within the left ocular globe (Figure 1). Ophthalmologist recommended no acute intervention and out-patient follow-up in two weeks.

**Figure 2:**
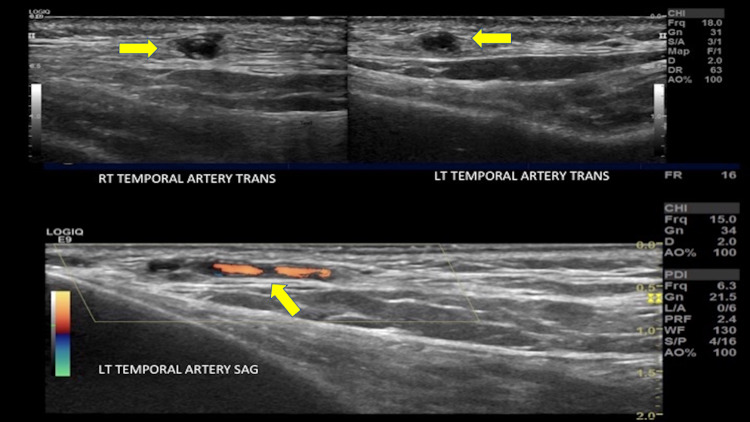
Ultrasound Doppler of Temporal Arteries demonstrating bilateral hypo-echoic thickening known as "Halo sign" (yellow arrowheads) suggestive of Temporal Arteritis.

Patient was admitted for pyrexia of unknown origin. She was noted to be febrile with oral temperature of 100F and leukocytosis of 16.6 x103/uL (Reference range 3.9-10 x10^3^/uL). Her absolute neutrophil count was elevated, along with lymphopenia but no bandemia was present. She also had transaminitis. Chest X-ray showed no acute pulmonary disease. Blood cultures were drawn and empiric broad-spectrum intravenous antibiotics, vancomycin and ceftriaxone, were initiated. Patient continued to spike fevers and had persistent leukocytosis over the next week. Retinal detachment remained a perplexing enigma. Blood cultures and urine cultures came back negative. CT Chest Abdomen Pelvis with contrast did not show any evidence of infection or overt malignancy. Full infectious work-up including tick-borne disease, parasites smear, influenza A/B, Cytomegalovirus and Epstein Barr Virus were all found to be negative. Rheumatologic workup ensued. Patient had significantly elevated erythrocyte sedimentation rate (ESR) of 120 mm/hr and c-reactive protein (CRP) 239 mg/L, antinuclear antibody (ANA) was positive with 1:160 speckled pattern. Patient was noted to have palpable, cord-like vessels in the temporal area bilaterally so Doppler ultrasound (US) of temporal arteries was ordered. The rest of the rheumatologic work-up including c-antineutrophil cytoplasmic antibodies (ANCA), p-ANCA, myeloperoxidase (MPO), proteinase 3 (PR3), and C4 were all within normal limits except C3 which was elevated. Doppler US of the temporal arteries revealed bilateral hypoechoic thickening (0.06cm) known as "halo sign" consistent with giant cell arteritis (Figure 2).

**Figure 3:**
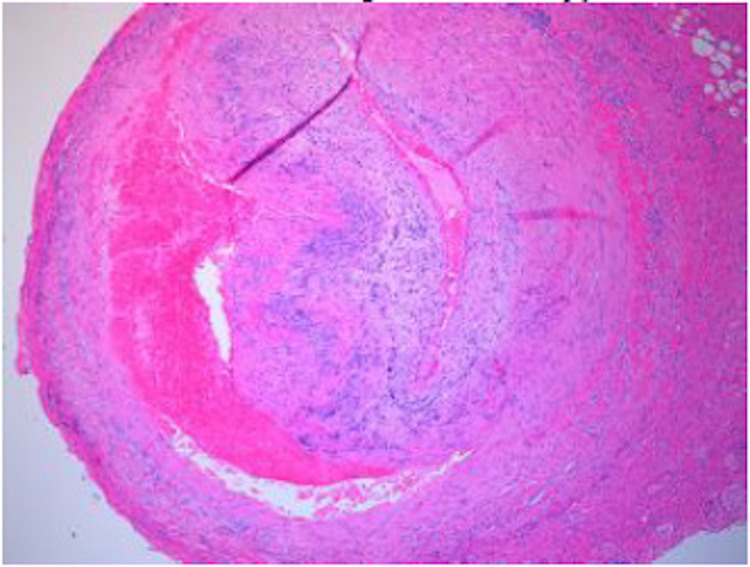
Temporal Artery Biopsy showing focal disruption of the internal elastic lamina with scattered inflammatory cell infiltrates and dissection of the mid-layer of the temporal artery with hemorrhage and compression of the arterial lumen confirming Temporal Arteritis with Dissection

Confirmatory left-sided temporal artery biopsy was performed which demonstrated focal disruption of the internal elastic lamina with scattered inflammatory cell infiltrates. While there was no granulomatous inflammation or giant cells, immunohistochemical stain for CD163 showed transmural histiocyte infiltration. In addition to these findings, there was also dissection of the mid-layer of the temporal artery with hemorrhage and compression of the arterial lumen, findings consistent with temporal arteritis with dissection (Figure 3).

Patient was started on pulse dose intravenous methylprednisolone 500mg daily for three days. After initiation of steroid therapy, patient began reporting immediate improvement in headaches and partial vision improvement as well. This was accompanied by resolution of fever and leukocytosis. Parenteral steroids were transitioned to prednisone 60mg PO daily for four weeks. She was discharged with an extensive tapering regimen and was scheduled to follow up outpatient with rheumatology and ophthalmology.

## Discussion

The diagnosis of GCA can be very challenging in acute patient care settings given the wide range of presenting symptoms and the possibility of delay in diagnosis due to the anchoring effect. It is imperative to consider GCA when ocular symptoms arise given the risk of permanent vision loss. Classification criteria for GCA issued by the American College of Rheumatology in 1990 can be helpful as a guide to consider GCA as the likely diagnosis or to differentiate it from other forms of vasculitis [5]. Studies have demonstrated that starting treatment prior to biopsy does not significantly reduce positive biopsy results, therefore steroid therapy should not be withheld [6].

The underlying etiology of GCA is complex and has been widely researched yet is still not well understood. This includes genetic and possibly infectious factors, which go on to trigger an immune response [7]. GCA is an antigen‐driven disease with local T cell and macrophage activation in the vessel wall, and with an important role of proinflammatory cytokines [8]. Inflammation of the arterial wall and vessel occlusion through fast and concentric intimal hyperplasia leads to the severe ischemic complications observed in patients with GCA [9]. 

Retinal vasculitis is an inflammatory response in the retinal blood vessels with inflammation extending towards nonvascular retinal structures which can result in retinal ischemia and rarely retinal detachment [10]. Dissection of the temporal artery is thought to be either due to the disease process causing vessel wall weakness or could be related to repeated palpation of the inflamed artery [11]. So far only one case of retinal detachment revealing GCA has been reported [12]; ours being the second one. There are a couple of case reports on temporal artery dissection [11] as a complication of GCA but our case is the first one presenting as a combination with both complications.

This case also emphasizes the role of Doppler US in evaluation of temporal arteritis compared to more costly imaging modalities. Studies have shown that US more frequently detects vasculitic changes in the large arteries compared to contrast-enhanced Magnetic Resonance Angiogram (MRA). When evaluating the cranial vessels, US performs similarly to MRI. This supports the recommendation that US be considered as a first-line evaluation in patients suspected to have GCA [13].

Corticosteroids remain the mainstay of treatment. If there is a strong suspicion of GCA as the cause of visual symptoms or signs, it is suggested to use intravenous "pulses" of methylprednisolone, administered as 500 to 1000 mg intravenously each day for three days, followed by oral therapy with prednisone 1 mg/kg/day (maximum of 60 mg/day), as recommended for uncomplicated GCA. The starting dose of high-dose prednisone is to be maintained for at least two, but not more than four, weeks. Although symptoms are typically controlled promptly by therapy, disease flares are the rule if the glucocorticoids are tapered too quickly. If the initial dose of prednisone is 60 mg/day, it can generally be reduced to 50 mg/day after two weeks and to 40 mg/day at the end of four weeks, assuming symptoms and signs have receded and the ESR and CRP have declined to normal or near-normal ranges. Subsequently, the dose can gradually be reduced by 5 mg every two weeks to 20 mg/day and then by 2.5 mg every two weeks to 10 mg/day if there are no flares of disease activity. After achieving a daily dose of 10 mg, the prednisone taper should be slowed, such that patients remain on progressively decreasing doses over the ensuing six to 12 months. Tapering by 1 mg decrements each month once the daily dose is less than 10 mg can be considered. Adjunctive treatment for giant cell arteritis (GCA) may be used in situations where glucocorticoid-related toxicities have ensued or are anticipated. Options include tocilizumab (TCZ) or methotrexate (MTX) [10,14].

Follow-up with rheumatology is crucial as there is substantial risk of relapses and also development of aortic aneurysm/dissection if left untreated [15]. Monitoring of disease activity requires close patient follow-up and monitoring of acute-phase reactants. 

## Conclusions

Retinal detachment and vitreous hemorrhage can be rare atypical manifestations of GCA. This may or may not be accompanied by temporal artery dissection which is another uncommon occurrence. Fever of unknown origin may be the presenting manifestation in some elderly patients with GCA. Doppler US should be considered as the first-line imaging modality to evaluate for temporal arteritis. Timely intervention with systemic glucocorticoids can prevent vision loss and promptly improve signs and symptoms of the disease. Providers need to stay vigilant and beware of anchoring bias when confronted with similar findings in this patient population.

## References

[1] Gonzalez-Gay MA, Vazquez-Rodriguez TR, Lopez-Diaz MJ, Miranda-Filloy JA, Gonzalez-Juanatey C, Martin J, Llorca J (2009). Epidemiology of giant cell arteritis and polymyalgia rheumatica. Arthritis Rheum.

[2] Rahman W, Rahman FZ (2005). Giant cell (temporal) arteritis: an overview and update. Surv Ophthalmol.

[3] Miller NR (2001). Visual manifestations of temporal arteritis. Rheum Dis Clin North Am.

[4] Weyand CM, Liao YJ, Goronzy JJ (2012). The immunopathology of giant cell arteritis: diagnostic and therapeutic implications. J Neuroophthalmol.

[5] Hunder GG, Bloch DA, Michel BA (1990). The American College of Rheumatology 1990 criteria for the classification of giant cell arteritis. Arthritis Rheum.

[6] Birkhead NC, Wagener HP, Shick RM (1957). Treatment of temporal arteritis with adrenal corticosteroids; results in fifty-five cases in which lesion was proved at biopsy. J Am Med Assoc.

[7] Pineles SL (2021). Giant Cell Arteritis. https://eyewiki.aao.org/Giant_Cell_Arteritis.

[8] Weyand CM, Hicok KC, Hunder GG, Goronzy JJ (1994). Tissue cytokine patterns in patients with polymyalgia rheumatica and giant cell arteritis. Ann Intern Med.

[9] Weyand CM, Goronzy JJ (1999). Arterial wall injury in giant cell arteritis. Arthritis Rheum.

[10] Docken WP (2021). Treatment of GCA.

[11] Kunst CH, Weiss ET, Klickstein LB (2001). Dissection of the temporal artery in a patient with giant cell arteritis. J Clin Rheumatol.

[12] Ouaggag B, Hajji I, Rajaa B, Baha AT, Moutaouakil A (2011). [Exsudative retinal detachment indicative of a giant cell arteritis. A case report]. Bull Soc Belge Ophtalmol.

[13] Yip A, Jernberg ET, Bardi M (2020). Magnetic resonance imaging compared to ultrasonography in giant cell arteritis: a cross-sectional study. Arthritis Res Ther.

[14] Delis A, Pollard CM, Prasad A, Sobonya RE, León LR Jr (2009). Isolated superficial temporal artery dissection masquerading as giant cell arteritis. Vascular.

[15] Bengtsson BA, Malmvall BE (1981). The epidemiology of giant cell arteritis including temporal arteritis and polymyalgia rheumatica. Incidences of different clinical presentations and eye complications. Arthritis Rheum.

